# Comparative Morphology of the Mouthparts in Three Predatory Stink Bugs (Heteroptera: Asopinae) Reveals Feeding Specialization of Stylets and Sensilla

**DOI:** 10.3390/insects11110762

**Published:** 2020-11-05

**Authors:** Yan Wang, Jolanta Brożek, Wu Dai

**Affiliations:** 1 Key Laboratory of Plant Protection Resources and Pest Integrated Management of the Ministry of Education, College of Plant Protection, Northwest A&F University, Yangling 712100, China; wang-yan@nwafu.edu.cn; 2 Faculty of Natural Science, Institute of Biology, Biotechnology and Environmental Protection, University of Silesia in Katowice, Bankowa 9, 40-007 Katowice, Poland; jolanta.brozek@us.edu.pl

**Keywords:** predatory stink bugs, stylet, sensilla, fine morphology

## Abstract

**Simple Summary:**

As the only predatory group in the family Pentatomidae, Asopinae are a diverse group of specialized soft-bodied insect predators, which have the potential for use in controlling pests of orchards, forests, and field crops. However, the feeding behavior remains poorly known for Asopinae, especially how the mouthpart structures relate to various functions in feeding. The fine structure of the mouthparts, including distribution and abundance of receptor sensilla, was observed in three species of Asopinae using scanning electron microscopy and structural details are described for the first time. The morphology of the mouthparts is similar to those of other Heteroptera. The four-segmented labium and labrum in all studied species have fourteen types of sensilla. Unlike phytophagous pentatomids, two types of olfactory sensilla with nanopores (St1, Sb3) were observed in these three predatory insects, which probably function to locate prey by smell. In predatory stink bugs, each mandibular stylet tip has five irregular teeth and three long, pointed hooks; the apices of the right maxilla have small teeth and few short barbs along the edge of the food canal. The structure and function of the mouthparts are adapted for predatory feeding in the three studied species. The detailed structure of the predatory pentatomid’s mouthparts shown in this study can provide more data for the morphological differentiation of the mouthparts in the future.

**Abstract:**

Mouthpart structures were observed in three species of Asopinae using scanning electron microscopy to investigate their morphological disparity. The examined species attack mainly slow-moving, soft-bodied insects, primarily larval forms of the Lepidoptera, and are the natural enemies of many pests. This is the first detailed description of their external mouthparts. The triangular and elongated labrum and four-segmented tube-like labium are longer in *Picromerus* species (*Picromerus bidens* (Linnaeus, 1758) and *Picromerus lewisi* Scott, 1874 than in *Cazira bhoutanica* Schouteden, 1907. The labrum of *P. lewisi* and *C. bhoutanica* appear to be equipped with olfactory sensilla basiconica Sb3, a special type of sensilla with nanopores. The labium surface in all studied species bears 14 types of sensilla (St1–St4, Sb1–7, Sst, Sca1–2). A new characteristic of sensilla trichodea is represented in sensillum St1; in both *Picromerus* species, it is classified as an olfactory sensillum with nanopores. The tripartite apex of the labium consists of two lateral lobes and a central membranous lobe having microtrichial extensions. Each lobe has one sensory field, including sensilla basiconica (Sb7), sensilla styloconica (Sst), and sensilla trichodea (St4). In the three studied predatory stink bugs, each mandibular stylet tip has five irregular teeth and three long, pointed hooks. The two opposing maxillae, which are held together by a tongue-and-groove system, form a food canal and a salivary canal. The apices of the right maxilla have small teeth and few short barbs along the edge of the food canal. In *P. bidens* and *P. lewisi*, there are 5 teeth, while in *C. bhoutanica* there are 2. Based on structural differences, we inferred that the hook-shaped mandibular teeth, right maxilla with small teeth, and few short barbs along edge of the food canal are more adapted for a predatory lifestyle. Predatory stink bugs use sharp recurved hooks and irregular teeth penetrating, tearing, or filing devices that aid in the mechanical disruption of host tissue. Stiff bristles in the food canal may indicate their possible adaptation to feeding on insect larvae. The evolution of mouthpart morphology and the putative functional significance of sensilla are discussed, providing insight into the sensory mechanism.

## 1. Introduction

The mouthparts of insects display a wondrous diversity of form and function. As feeding structures, mouthparts play an important role in the evolution of insects. The structure of mouthparts varies according to the nature of their food. The structural differences are especially significant in the more derived orders and can be used as features for identification and classification [1,2,3]. During the feeding process, sensory organs on the surface of the mouthparts can identify and locate the host [4,5]. In Hemiptera, because the apex of the labium first comes into contact with the feeding site, the sensory structures on the labial tip are most concentrated and diverse. The types and quantity of sensilla are closely related to the feeding habits of the insect [6]. In addition to these sensory organs, the stylet fascicle is also very important for feeding. After the mandibular stylet is inserted into the host tissue, it is mainly used for fixation, providing support for the maxillary stylets, and helping penetrate deeper to feed [1]. The ridge on the outer surface of the proximal end of the mandibular stylet and the teeth on both sides contribute to the depth of puncture. During evolutionary diversification, the morphology of stylets became differentiated, with substantial differences emerging among different lineages [1,2,6,7,8,9,10,11,12,13,14,15,16,17,18,19,20,21,22]. Most prior studies of hemipteran mouthparts only focused on one aspect, the sensilla on the labium tip or the stylet fascicle, and detailed study of the mouthparts overall is often lacking. More comprehensive studies are needed in order to provide useful comparative data.

Heteroptera is one of the most abundant groups of hemimetabolous insects and includes many economically important taxa, such as agricultural pests, animal parasites, and natural enemies of pest insects. Pentatomidae is one of the most common large families in Heteroptera and members are found worldwide. While most members of Pentatomidae are phytophagous, most Asopinae are predatory, preying on Coleoptera and Lepidoptera larvae [23]. Some species of this group have been used in biocontrol of agricultural pests [23]. Several prior studies reported on the morphology of the mouthparts of Pentatomidae [1,8,24,25,26,27,28]. Most of these studies focused on the tip of the labium [26,27], stylets [1,8,12,13] and the general structure of mouthparts [24,25,28]. Among them, only four predatory pentatomids were studied: *Perillus bioculatus* (Fabricius) by Cobben [1] and Parveen et al. [27]; *Podisus maculiventris* (Say) by Cohen [8]; *Eocanthecona furcellata* (Wolff) by Rani [26] and Parveen et al. [27]; and *Canthecona furcellata* (Wolff) by Barsagade and Gathalkar [28]. More species and more detailed information on fine structure are needed to determine how much variability in mouthparts occurs among pentatomid groups with different feeding habits.

The subfamily Asopinae, commonly called predatory stinkbugs or soldier bugs, has more than 300 species in 63 genera worldwide [29] and most of the genera and species are distributed in the trans-Palearctic [30,31,32]. The main synapomorphy of this subfamily is the stout rostrum, which is an adaptation for predation on other insects [33]. Predatory stinkbugs inhabit forests and grasslands as well as agroecosystems such as orchards and vegetable fields. Many species appear to prefer shrubland and forests. As the only predatory group in the family Pentatomidae, Asopinae attack mainly slow-moving, soft-bodied insects, primarily larval forms of Lepidoptera, and are the natural enemy of pests (e.g., Colorado potato beetle and Mexican bean beetle) [23]. The predatory pentatomids, therefore, have potential for use in in controlling pests of orchards, forests, and field crops [34,35,36].

This study explores the sensory structures, both maxillary and mandibular, of three species of Asopinae, belonging to two genera, *Picromerus bidens* (Linnaeus, 1758), *Picromerus lewisi* Scott, 1874, and *Cazira bhoutanica* Schouteden, 1907, which are all predatory. *P. bidens* is distributed in forests and grasslands of China (Anhui, Hunan, Hebei, Heilongjiang, Jilin, Liaoning, Inner Mongolia), Europe, and northeastern North American [23,37,38]. It is a highly polyphagous bug that feeds mainly on leaf-feeding larvae of the Lepidoptera, Coleoptera, and Hymenoptera [23]. Prior studies have reported that this species has the potential to reduce the number of pests in various ecosystems [23,39,40,41,42,43,44,45,46,47,48,49]. *P. lewisi* is found in China, Japan, Korea, and Russia [37,50,51], and feeds on Lepidoptera, Diptera and other pests [52,53]. In Yanbian, Jilin, it may attack the larvae of tussah silkworms, and in Tonggu, Jiangxi, it is found to injure tea. *C. bhoutanica* is distributed in China (Jiangsu, Anhui, Zhejiang, Jiangxi, Fujian, Sichuan, Guizhou, Yunnan), India, Nepal, and Bhutan [50]. This bug mainly preys on cotton aphids. Prior research on these three stink bugs focused on taxonomy and bionomic characteristics. There are no detailed reports on their mouthparts.

For this study, we have three aims: (1) to provide the first detailed fine morphological characterization of the mouthparts of three Asopinae species using scanning electron microscopy (SEM); (2) to show how the mouthparts of these predatory pentatomids differ from those of phytophagous pentatomids (incorporating data from references); and (3) to determine what roles the mouthpart structures (labrum, labium, sensilla, mandibular, and maxillary stylets) play in their feeding process.

## 2. Material and Methods

### 2.1. Insect Collecting

The specimens (n = 7) of Asopinae used in this study were collected in China and Poland. *P. bidens* was collected by Yan Wang in Szczawnica, Poland (49°43′ N, 20°50′ E, elev. 674 m) in August 2019 and preserved in 95% ethanol. *P. lewisi* was collected by Chao Wen in Fanjing Mountain, Guizhou Province, China (27°913′ N, 108°694′ E, elev. 2572 m), in July 2016 and preserved in 95% ethanol. *C. bhoutanica* was collected by Wang Yan in Siming Mountain, Ningbo, Zhejiang Province, China (29°75′ N, 121°90′ E, elev. 823 m), in July 2015 and preserved in 95% ethanol.

### 2.2. Samples for SEM

The sampled specimens (n = 7) were dipped into 10% NaOH solution for 2 h and cleaned twice by using an ultrasonic cleaner (KQ118, Kunshan, China) for 15s each time. Dehydration involved serial baths of 80%, 90% and 100% ethanol each for 15 min. The specimens were air dried, coated with a film of gold (Q150T-S, Quorum, West Sussex, UK), and then imaged with a Nova Nano SEM-450 (FEI, Hillsboro, OR, USA) at 5 kV in the scanning microscopy laboratories of the Life Science Research Core Services of Northwest A & F University, Yangling, Shaanxi, China.

### 2.3. Image Processing and Morphometric Measurement

SEMs were observed and measured after being imported into Adobe Photoshop CC 2019 (Adobe Systems, San Jose, CA, USA). Statistical analyses were executed using SPSS 19.0 (SPSS, Chicago, IL, USA). Graphs were fitted using Microsoft Office Excel 2007.

### 2.4. Terminology

The sensilla were classified according to their external morphology, length, distribution, and position. The terminology of sensilla follows Altner and Prillinger [54] and Frazier [55] with less specialized nomenclature from Parveen et al. [27].

## 3. Results

### 3.1. Overall Morphology of the Mouthparts

**Figure 1:**
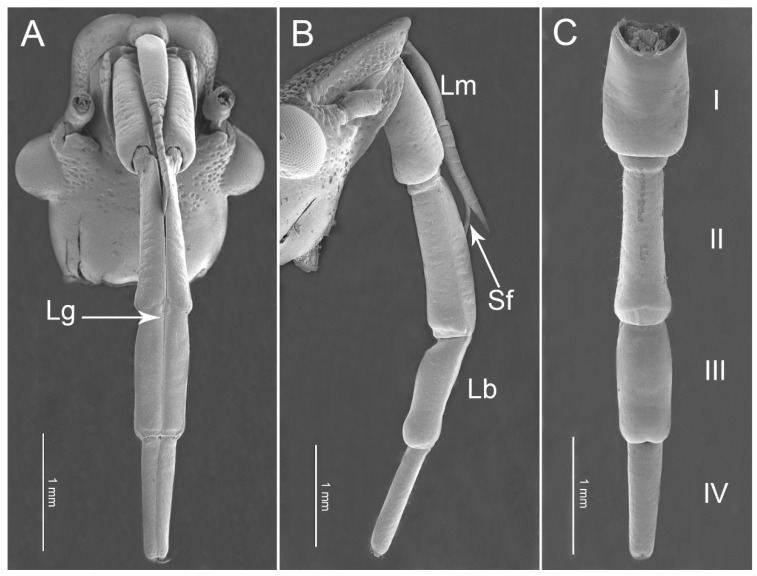
Scanning electron micrographs of the head of *Picromerus lewisi*. (**A**) Ventral view; (**B**) Lateral view; (**C**) Dorsal view showing four-segmented labium (I–IV); Lg, labial groove; Lm, labrum; Lb, labium; Sf, stylet fascicle.

The mouthparts of *P. bidens*, *P. lewisi*, and *C. bhoutanica* are similar to those of other true bugs, consisting of a tapered labrum (Lm), a long four-segmented labium (Lb), and a pair of separated mandibular and interlocked maxillary stylets, which are enclosed in the groove of the labium (Figure 1A–C). When the insect is resting or not feeding, the mouthparts (rostrum) are pressed to the sternum, parallel to the body.

#### 3.1.1. Labrum

**Figure 2:**
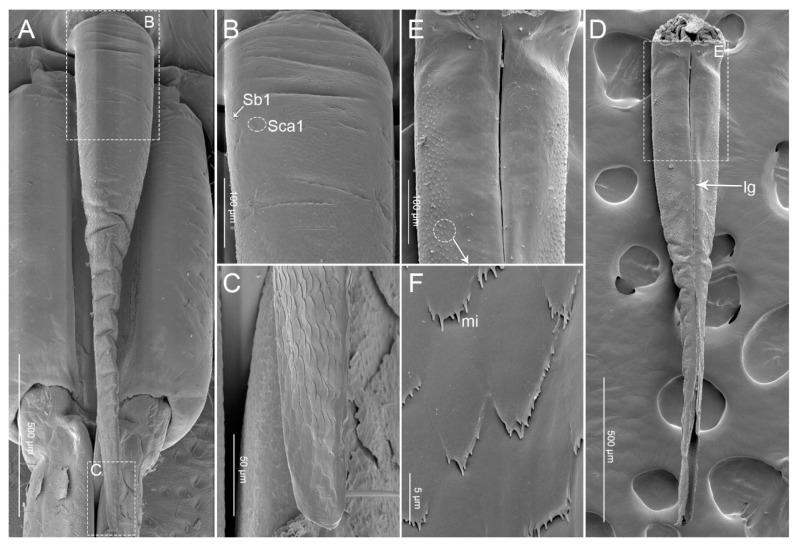
SEM images of labrum of *Picromerus bidens*. (**A**) Ventral view; (**B**,**C**) Enlarged view of box in (**A**), showing sensilla basiconica (Sb1) and sensilla campaniformia Sca1; (**D**) Dorsal view; (**E**) Enlarged view of box in (**D**); (**F**) Clusters of microtrichia (mi); lg, labral groove.

**Figure 3:**
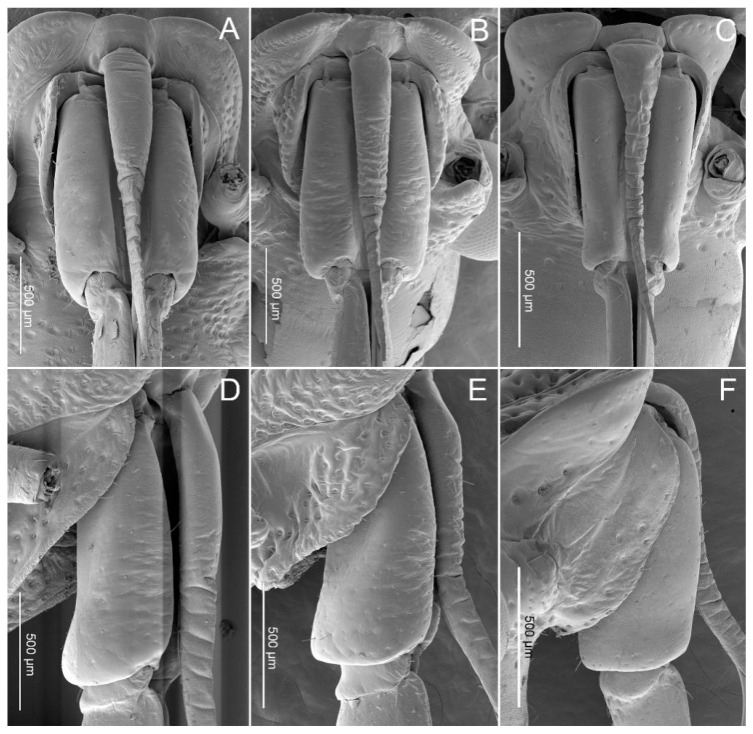
SEM images of the labrum and first labial segment. (**A**) Ventral view of *Picromerus bidens*; (**B**) Ventral view of *Picromerus lewisi*; (**C**) Ventral view of *Cazira bhoutanica*; (**D**) Lateral view of *Picromerus bidens*; (**E**) Lateral view of *Picromerus lewisi*; (**F**) Lateral view of *Cazira bhoutanica.*

**Figure 4:**
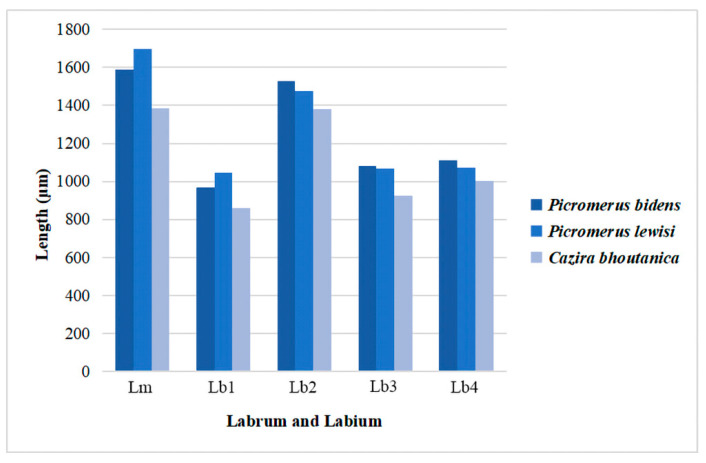
The length of labrum and each labial segment in different species. Lm, labrum; Lb1, 2, 3, and 4: the first, second, third, and fourth labial segments, respectively.

The labrum (Lm) is triangular, elongated, with the base connected to the anteclypeus (Figure 2A and Figure 3A–C). In *P. bidens*, the basal half is wide and relatively smooth, while the distal half is extremely narrow and wrinkled. In *P. lewisi*, the basal quarter is relatively smooth, the distal three quarters are strongly plicated. However, in *C. bhoutanica*, the basal quarter is relatively smooth while the distal four quarters are strongly plicated. The lengths of the labrum are 1586.8 µm in *P. bidens*, 1694.8 µm in *P. lewisi*, and 1385.9 µm in *C. bhoutanica*, respectively. In all three species, the labrum is longer than the first labial segment and completely covers the groove of the first labial segment (Table 1, Figure 3A–C and Figure 4). The ventral side is covered with some sensilla basiconica Sb1, Sb2, and Sb3 (Table 2, Figure 2B). Sensilla basiconica I (Sb1) are present in *P. bidens*, *P. lewisi*, and *C. bhoutanica*; sensilla basiconica II (Sb2) occur only in *P. lewisi*; and sensilla basiconica III (Sb3) are found in *P. lewisi* and *C. bhoutanica*. According to their external morphology (not porous and embedded in flexible sockets), Sb1 and Sb2 represent mechanosensilla (Table 3). The surfaces of sensilla basiconica Sb3 are slightly porous and several very small pores (diameters of these nanopores approximately 31.6 ± 2.0 nm (n = 5)) are present. Many scale-like projections cover the ventral surface of the labrum (Figure 2A–C). On the dorsal side of the labrum is the conspicuous labral groove (lg) together with a strongly sclerotized hypopharyngeal lobe forming a trough for the stylet bundle (Figure 2D,E). Clusters of microtrichia (mi) are arranged in irregular transverse rows on the dorsal area of the labrum (Figure 2D–F).

#### 3.1.2. Labium

The labium (Lb) (Figure 1A–C) is elongated to form a protective sheath within which the stylet bundle is enclosed. In the studied species, the first (I), second (II), third (III), and fourth segments (IV) have a similar shape but differ in length (Table 1, Figure 4). Based on the surface sculpture and pore system, socket form, shaft shape, and length, fourteen subtypes of sensilla were observed on the surfaces of the labium (Table 2 and Table 3).

**Figure 5:**
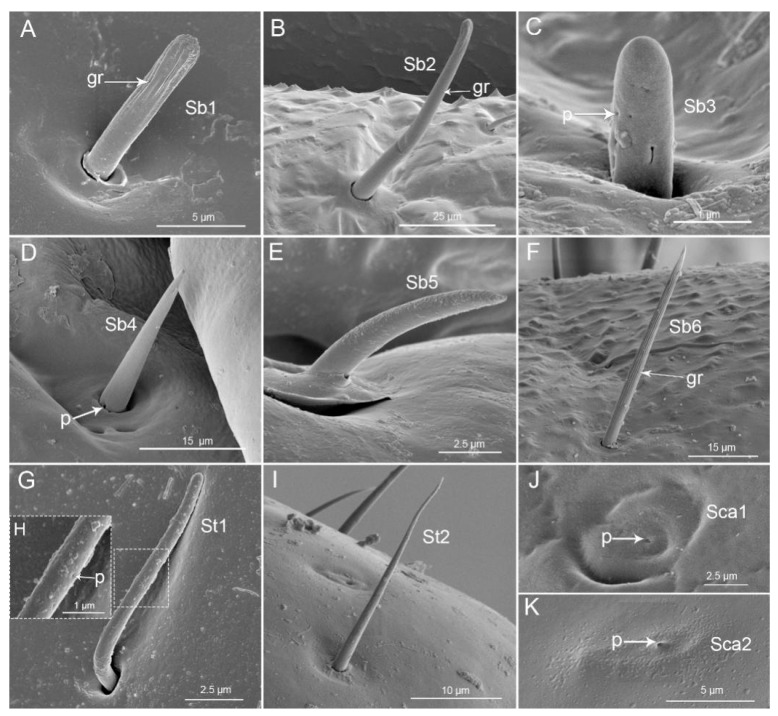
SEM images of sensilla of three species. (**A**) Sensilla basiconica I (Sb1); (**B**) Sensilla basiconica II (Sb2); (**C**) Sensilla basiconica III (Sb3); (**D**) Sensilla basiconica IV (Sb4); (**E**) Sensilla basiconica V (Sb5); (**F**) Sensilla basiconica VI (Sb6); (**G**) Sensilla trichodea I (St1); (**H**) Enlarged view of sensilla trichodea I (St1); (**I**) Sensilla trichodea II (St2); (**J**) Sensilla campaniformia I (Sca1); (**K**) Sensilla campaniformia II (Sca2); gr, groove; p, pore.

In these three species, the first (basal) segment (I) is short and thick, surrounded and partially covered by the small low bucculae, but the other segments are clearly exposed (Figure 3A–F). The first segment is the shortest segment of the labium in these species (Figure 4). On the ventral surface, the labial groove (Lg) is very wide and open enough to accommodate the labrum (Figure 3A–C). The distal ventro-lateral part of the segment is slightly widened (Figure 3D–F). Sensilla found on this segment are as follows: Sensilla trichodea I (St1) are hair-like, with a curved and round apex and are inserted in a pit (Figure 5G) in *P. bidens* and *P. lewisi*. The surfaces of these sensilla are covered by nanopores (diameters of approximately 0.0587µm = 58.7 ± 4.3 nm (n = 8)) but visible only at very high magnification. These sensilla are positioned solitarily on the surface of the first segment. Sensilla basiconica I (Sb1) that are short, straight, robust, and have a grooved surface and flexible socket (Figure 5A) are present in all the studied species. Sensilla basiconica II (Sb2) are longer with a curved, grooved surface and a flexible socket (Figure 5B). This type of sensilla occurs in *P. bidens* and *C. bhoutanica*. Sensilla basiconica III (Sb3) are similar to sensilla coeloconica, being short cones that arise from inflexible sockets (diameter of these nanopores on Sb3 is 31.6 ± 2.0 nm (n = 5)) (Figure 5C). This type of sensillum is present in *C. bhoutanica*. Sensilla basiconica V (Sb5) are conical, curved, and robust with a smooth surface (Figure 5E). This type of sensillum is found in *P. bidens* and *P. lewisi*. Sensilla basiconica VI (Sb6) are very long, straight, and almost perpendicular to the surface of the labium (Table 2, Figure 5F). The bases of these sensilla have a flexible socket, the surface with a vertical groove, and the tip is narrow (Figure 5F). This type of sensilla is only found in *P. bidens*.

**Figure 6:**
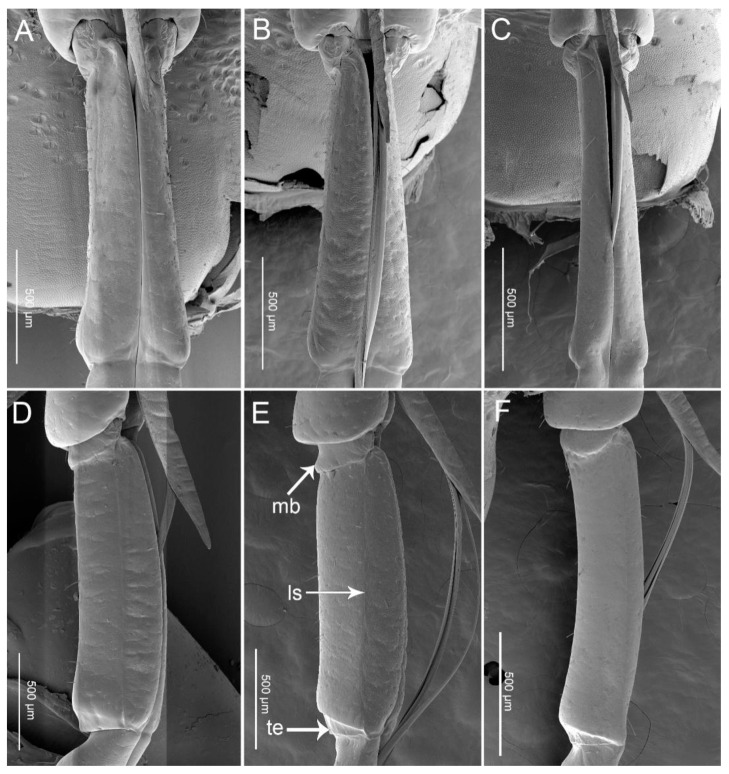
SEM images of the second labial segment. (**A**) Ventral view of *Picromerus bidens*; (**B**) Ventral view of *Picromerus lewisi*; (**C**) Ventral view of *Cazira bhoutanica*; (**D**) Lateral view of *Picromerus bidens*; (**E**) Lateral view of *Picromerus lewisi*; (**F**) Lateral view of *Cazira bhoutanica*; mb, membrane; ls, longitudinal suture; te, tapered edge.

The second segment (II) is the longest segment of the labium (Figure 4). In the ventral view, the second segment gradually widens from base to apex (Figure 6A–C); in the lateral view, however, it is of uniform width throughout most of its length (Figure 6D–F). The base of this segment forms a distinct articulation with the first segment, consisting of a band-like dorsal plate and a ventro-lateral band of membrane (mb) (Figure 6D–F). A longitudinal suture (ls) extends along each side of the second segment (Figure 6D–F). The distal part of the second labial segment has a tapered edge (te) with a lateral membrane (Figure 6D–F). There are three pairs of sensilla basiconica (Sb4) at the base of the second segment of all three species. They are of medium length, straight, with a smooth surface, and have a basal wall pore (molting pore), a rounded tip, and a flexible socket (Table 2, Figure 5D). Aside from sensilla basiconica IV (Sb4), there are six types of sensilla (St1, Sb1, Sb2, Sb3, Sb5, Sca1) on the second segment that slightly differ in these three species (Table 2). Sensilla trichodea I (St1) have the same characters as on the first segment and are also visible in *P. bidens* and *P. lewisi*. Sb1 are only found in *P. bidens* and Sb3 in *C. bhoutanica* (the same as the Sb3 on the labrum). Sensilla basiconica II (Sb2), sensilla basiconica V (Sb5), and sensilla campaniformia I (Sca1) are distributed in *P. lewisi* and *C. bhoutanica*. Sca1 are cupola-shaped structures with a slightly convex central part and with a central pore (molting pore) (Figure 5I).

**Figure 7:**
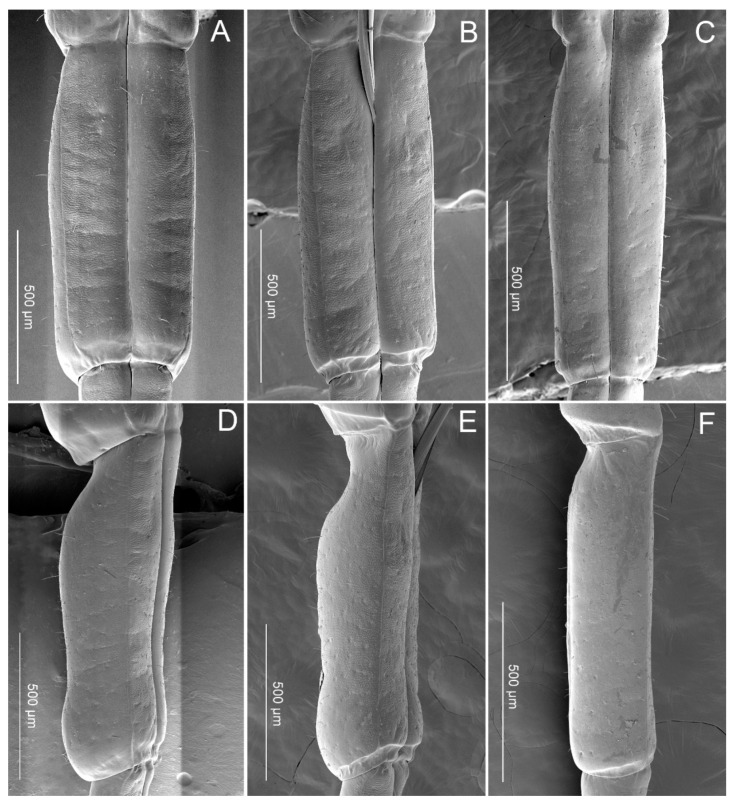
SEM images of the third labial segment. (**A**) Ventral view of *Picromerus bidens*; (**B**) Ventral view of *Picromerus lewisi*; (**C**) Ventral view of *Cazira bhoutanica*; (**D**) Lateral view of *Picromerus bidens*; (**E**) Lateral view of *Picromerus lewisi*; (**F**) Lateral view of *Cazira bhoutanica.*

The third segment (III) is shorter than the second segment and is nearly uniform in width through most of its length (Figure 7A–C). In lateral view, the base is constricted (Figure 7D–F). A longitudinal suture (ls) also extends through each side of this segment. There are fewer sensilla distributed on this segment. St1 are present in small quantities in *P. bidens* and *P. lewisi*. Sb3 are only found in *C. bhoutanica*, Sb5 in *P. bidens* and Sb6 in *P. lewisi*.

**Figure 8:**
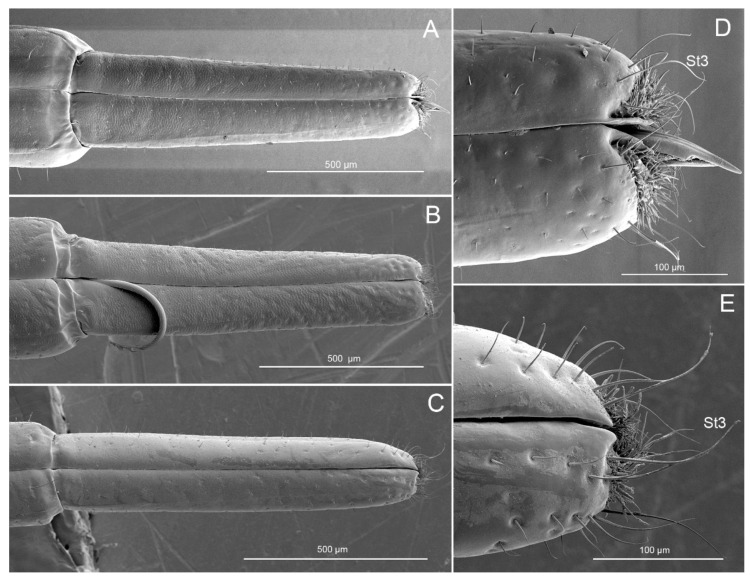
SEM images of ventral view of the fourth labial segment. (**A**) Ventral view of *Picromerus bidens*; (**B**) Ventral view of *Picromerus lewisi*; (**C**) Ventral view of *Cazira bhoutanica*; (**D**) Apex of the fourth labial segment of *Picromerus bidens*; (**E**) Apex of the fourth labial segment of *Cazira bhoutanica,* St3, sensilla trichodea III.

The fourth, or distal labial, segment is short (Figure 4) and conical in shape (Figure 8A–C). A longitudinal suture (ls) is also found on each side of this segment. A large number of sensilla are present on the surface of this segment. One pair of Sb4 are present on each side of the junction of the third and fourth segments of the species. St1 are visible in *P. bidens* and *P. lewisi*. St2, St3, Sb4, Sb6, and Sca2 are found in these species. Sb1 are only distributed in *P. bidens,* Sb5 in *P. lewisi*, and Sca1 in *C. bhoutanica*. St2 arranged roughly on the apical 1/6 are smooth and slender, slightly curved in the apical half, and inserted in a flexible socket (Figure 5H). St3, arranged on the labial subapex, are long and slender, slightly curved, and smooth with a flexible socket (Figure 8D,E). Several Sca2 are located on the antero-lateral surface near the apical 1/5. Each is a sunken circular plate with a central pore (Figure 5J).

**Figure 9:**
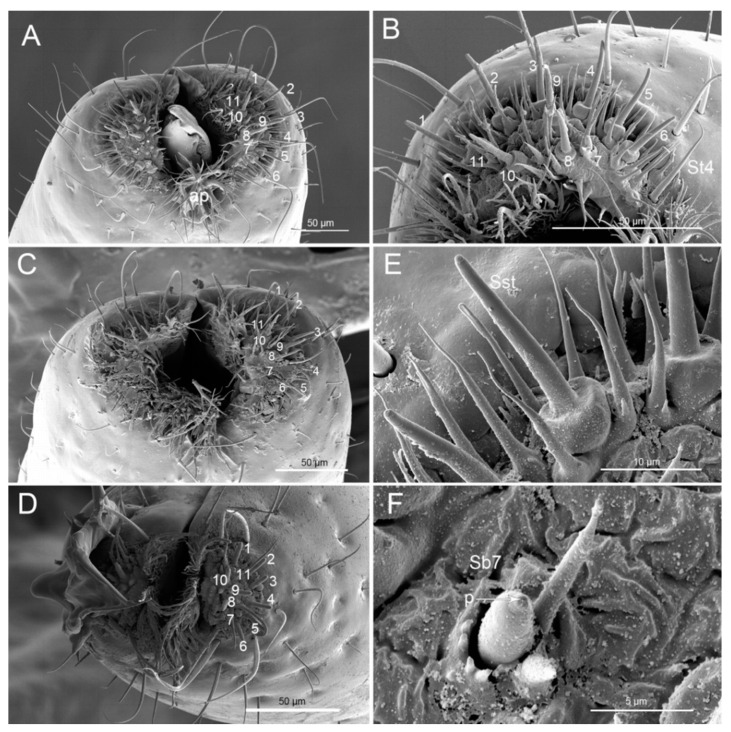
SEM images of the tip of the labium. (**A**) Vertical view of the tip of *Picromerus bidens*; (**B**) Right side of the tip of *Picromerus bidens* showing sensilla basiconica VII (Sb7) (nos. 7 and 11), sensilla styloconica (nos. 1–6, 8–10) and sensilla trichodea IV (St4); (**C**) Vertical view of the tip of *Picromerus lewisi*; (**D**) Vertical view of the tip of *Cazira bhoutanica*; (**E**) Sensillum styloconicum (Sst); (**F**) Sensillum basiconicum (Sb7); p, pore.

The tripartile labial tip is divided into two lateral lobes and a central membranous lobe bearing microtrichial extensions (Figure 9A–F). Each lobe has one sensory field, including sensilla basiconica (Sb7) (nos. 7, 11), sensilla styloconica (Sst) (nos. 1–6, 8–10), and sensilla trichodea (St4). Nine Sst are present at the center of each lobe (Figure 9B). Sst are robust, straight, with a smooth surface, and are inserted on a raised platform (Figure 9E). Two Sb7 have an ovoid body, a socket surrounding the base of the shaft, and a partial hood formed by fused cuticular processes (Figure 9F). A mass of St4 are present at each lobe (Figure 9B). St4 are hair-like with a curved, pointed apex and smooth surface. Spines and comb-like structures are laterally situated within the stylet groove of the last labial segment, probably serving to clean the mandibles during and after feeding.

According to their morphology, putative functions of the aforementioned labial tip sensilla are presented in Table 3.

### 3.2. Stylet Fascicle

The stylet bundle consists of two separate mandibular stylets (Md) and two interlocked maxillary stylets (Mx). Usually, the mandibular stylets used for tearing are shorter than the absorbed maxillary stylets.

#### 3.2.1. Mandibles

**Figure 10:**
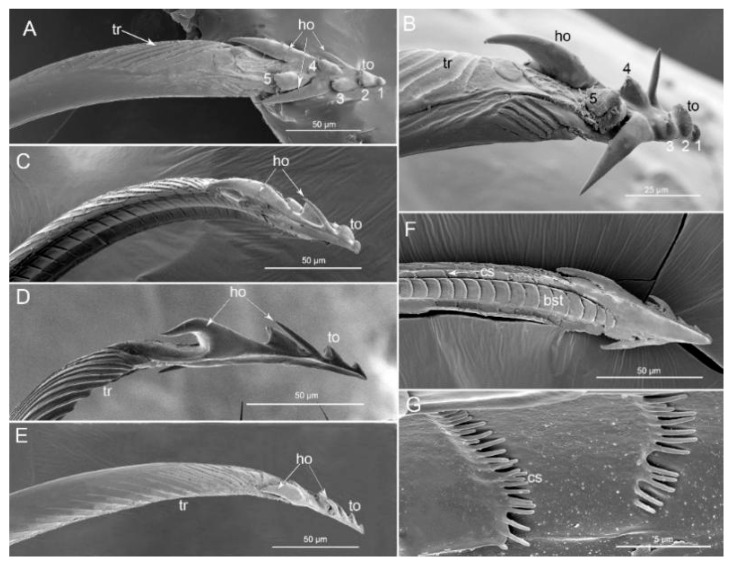
SEM images of mandibular stylets of three species. (**A**) External view of stylet in *Picromerus bidens* showing transverse ridges (tr), five irregular teeth (to) and three long and pointed hooks (ho); (**B**) External view of stylet in *Picromerus bidens*; (**C**) Lateral view of stylet in *Picromerus bidens*; (**D**) Lateral view of stylet in *Picromerus lewisi*; (**E**) Lateral view of stylet in *Cazira bhoutanica*; (**F**) Interior side of stylet of *Cazira bhoutanica* showing small cuticular spines (cs) and big squamous textures (bst); (**G**) Small cuticular spines (cs); 1–5, irregular teeth.

The mandibular stylets (Md) are attached to and surround the two interlocking maxillary stylets (Mx). The apical region, external, lateral, and internal parts of the mandibular (Md) stylets were observed in all three predatory stinkbugs (*P. bidens*, *P. lewisi,* and *C. bhoutanica*). The apices of the mandibles have a distinct curvature and bear rows of transverse ridges (tr) over much of their outer surfaces. Their apices are irregular with teeth (to) and long, pointed hooks (ho) (Figure 10A–E). In the three species of Asopinae (*P. bidens*, *P. lewisi*, and *C. bhoutanica*) two irregular teeth (to) are placed anteriorly. Three big irregular teeth (to) and three long, pointed hooks (ho) are placed dorso-laterally (Figure 10A,B). The mandibles have some longitudinal grooves over much of their inner surfaces that differ between the left and right in all studied species. The first row consists of small cuticular spines (cs) (Figure 10F,G). The second row possesses big squamous textures (bst) (Figure 10F). The inner surfaces of the ends of the mandibles are smooth (Figure 10F).

#### 3.2.2. Maxillae

**Figure 11:**
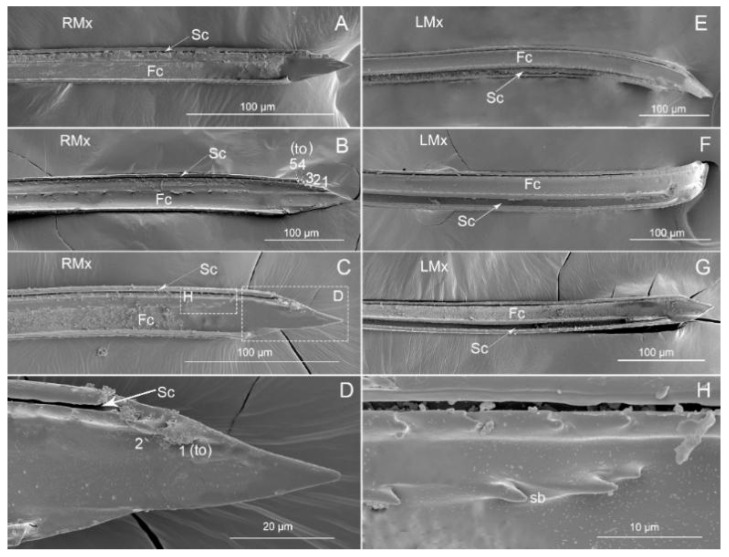
SEM images of maxillary stylets of three species. (**A**) *Picromerus bidens*; (**B**) *Picromerus lewisi*; (**C**) *Cazira bhoutanica*; (**D**) Enlarged view of box in (**C**); (**E**) *Picromerus bidens*; (**F**) *Picromerus lewisi*; (**G**) *Cazira bhoutanica*; (**H**) Enlarged view of box in (**C**); Fc, food canal; Sc, salivary canal; LMx, left maxillary stylet; RMx, right maxillary stylet; to, tooth; sb, short barbs; The number represents the number of teeth.

In these three species, maxillary stylets are equipped with a series of ridges and grooves internally and the left and right maxillary stylets are asymmetric. The two opposing maxillae, which are held together by a system of tongue-and-grooves, form a food canal and a salivary canal. The apex of the right maxilla (RMx) is straight with several ventral teeth (to) and ventral rows that have short barbs (sb) in the food canal (Figure 11A–D,H). In *P. bidens* and *P. lewisi*, the number of teeth (to) is 5, and there are 2 in *C. bhoutanica*. The apex of the left maxilla (LMx) is tapered and has a distinct curvature (Figure 11E–G). In our observation, the curvature *P. bidens* and *P. lewisi* is greater than that of *C. bhoutanica*.

## 4. Discussion

This study presents detailed observations of the mouthpart structures in three species of Asopinae (Heteroptera: Pentatomidae). This reveals some interesting morphological features and allows for morphological comparison as well as a better understanding of the feeding mechanism and the sensory system of these predatory stinkbugs when compared to phytophagous heteropterans (Table 4). Because the Asopinae are likely derived from plant-feeding ancestors [56], differences in the mouthparts of this group may reflect a shift from phytophagy to predation [57].

### 4.1. The Mouthparts of Predatory Specialist

Diagnostic characteristics of the Asopinae include, among others, the following modifications of the head associated with their feeding habits: rostrum strongly incrassate, the insertion of labium very close to base of labrum, and the posterior margins of buccula merged [33,58]. Because the labrum is frequently omitted in most studies as an element of the mouthparts, we included it in this study for structural and functional analysis.

The lengths of the labrums vary among the three species of predatory stinkbugs we observed (1586.8 µm in *P. bidens*, 1694.8 µm in *P. lewisi* and 1385.9 µm in *C. bhoutanica*), but they are consistently longer than the first segment of the labium (Table 1 and Figure 3). This is consistent with two other predatory stinkbugs, *Canthecona furcellata* (Wolff) [28] and *Apateticus cynicus* (Say) [59], in which a narrow elongate labrum rests over the proximal portion of the labial groove and extends distally through the first and part way into the second labial segment. The median part of the ventral surface of the labrum is slightly concave, forming a sort of trough holding the stylet bundle [59]. Based on our images of Asopinae species (Figure 3) and other information [59] it can be assumed that the long labrum may be important for predatory stinkbugs to hold the proximal part of the stylets together during feeding, preventing excessive bending in this part of the stylet bundle. The short membrane at the anterior basal portion of the labrum allows freedom of forward movement of the labrum from its normal concealed position beneath the head and controls the path of the stylet bundle. Comparison of similar-sized phytophagous pentatomid species (*Chinavia hilaris* (Say) vs. *Euschistus servus* (Say), and *Oebalus pugnax* (Fabricius) vs. *Piezodorus guildinii* Westwood) showed differences in lengths of the labial segments (Table 3, [60]) and their influence on depth of penetration of the stylets in host tissues, as well as the role of the labrum in feeding. However, the role of labrum size has not been demonstrated. Esquivel [60] described how, during probing, the labrum remains against the ventral surface of the head and the stylet bundle remains within segments 2, 3, and 4. However, when assuming a feeding position, the labrum swings away from the body and the stylet bundle emerges from the length of segment 2 [60]. We found more general information in prior publications on pentatomids on the total length of the labium (rostrum) than on the labrum. Nevertheless, a comparison of Asopinae with different phytophagous heteropteran bugs, e.g., *Pyrrhocoris sibiricus* (Kuschakevich) [16], *Cheilocapsus nigrescens* (Liu and Wang) [18], *Stephanitis nashi* (Esaki and Takeya) [19] and *Macrocheraia grandis* (Gray), *Physopelta quadriguttata* Bergroth, *Physopelta cincticollis* (Stål), and *Physopelta gutta* (Burmeister) [20] showed that the labrum is shorter than the first labial segment in these species. More data on the labrum length of phytophagous pentatomids is needed to facilitate further comparison.

The labium of these three predatory stinkbugs (*P. bidens*, *P. lewisi*, and *C. bhoutanica*) is four-segmented as in most other heteropteran bugs [16,18,19,20,28]. In these species, the base of the second segment forms a distinct articulation (band-like dorsal plate and ventro-lateral band of the membrane) with the first segment (Figure 6D–F). Such features have not been reported in previous studies of phytophagous bugs [16,18,19,20].

The lengths of the labial segments of the species studied here (Table 1) are different from another species of Asopinae, *Eocanthecona japanicola* (Esaki & Ishihara) (I—1120 µm, II—1430 µm, III—1220 µm, IV—1140 µm, cumulative length 4910 µm) [61]. Phytophagous Pentatomidae have similar relative proportions of individual labial segments to those found in Asopinae, but the cumulative length of the labial segments is significantly longer in phytophages (Table 3, [60]). In both groups, the first segment is shorter than the second and the third and fourth have a similar length but are shorter than the second. In asopines, the first segment of the labium (rostrum) is markedly thickened and free (not concealed between bucculae) [62], which enables the rostrum to swing forward fully, making it easier for the predator to feed on active prey [23]. This is probably an essential difference of the rostrum structure between asopines and other pentatomids. In the two groups, feeding stages differ slightly in the relative positions of labial segments. In phytophage feeding, the angle between the first and second segment is more acute [63] in Asopinae. This causes a shortening of the overall distance from the apex of the head to the distal end of the labium. This results in the stylet bundle exiting the distal end of the rostrum, thereby causing stylet penetration into the host [63]. In the Asopinae, the labium assumes a more prognathous position during feeding. The first and second segments also bend, but not as strongly, shortening the length of the labium to allow deep penetration of the stylets. The second segment (Figure 4) probably controls the activity of the short third and fourth segments.

The switch from a plant-feeding ancestor to a predaceous true bug likely occurred in the most recent common ancestor of all Heteroptera and the predatory life-style was retained in most lineages, although secondary transitions to phytophagy occurred, especially in the Pentatomomorpha and Cimicomorpha, with subsequent reversals to predation in some taxa [64]. The main functional elements of the mouthparts are mandibular and maxillary stylets, and their adaptive characters reflect trophic differences [1,65].

Different numbers and shapes of teeth on the mandibles in many heteropteran taxa have been reported by several authors [1,2,6,7,8,9,10,11,12,13,14,15,16,17,18,19,20,21,22,24,66,67]. Generally, Faucheux [2] commented that the number of mandibular teeth in heteropterans ranges from 4 to 40, with the number of teeth in phytophagous bugs significantly lower and less variable among different groups. The tips of mandibular stylets with teeth and hooks and their numbers and shapes are very similar among the studied species. In *P. bidens*, *P. lewisi*, and *C. bhoutanica*, two irregular teeth are placed anteriorly, three big teeth and three very pointed, long and recurved hooks are placed dorso-laterally (Figure 10). Cobben [1] suggested the predatory stink bug *Perillus bioculatus* Fabricius (Asopinae) uses sharp recurved hooks and irregular teeth as a penetrating, tearing or filing device that aids in the mechanical disruption of host tissues.

The abovementioned characters of the mandible in species studied so far correspond to Cobben’s [1] description of the mandibular tips in *Perillus bioculatus* Fabricius (Asopinae), which also have an inward curvature similar to those of typical phytophagous bugs. The mandibular stylet behavior of Perillus feeding on lepidopteran larvae is similar to that of seed-feeding bugs [1]; however, some special elements of the mandible structures are different. We documented in previous studies that mandibular structures of such predators differ from those of seed feeding bugs (three central blunt teeth and two pairs of lateral teeth in *Pyrrhocoris sibiricus* [16], and 1–2 teeth on the anterior and 1–2 dorso-lateral side in Largidae [20]). This very similar pattern of the mandible serration to seed-feeding bugs is present in phytophagous stink bugs. Depieri and Panizzi [12] reported that species *Dichelops melacanthus* (Dallas), *Euschistus heros* Fabricius, *Nezara viridula* (Linnaeus), and *Piezodorus guildinii* (Westwood) have four central blunt teeth and three pairs of small lateral teeth. This study of Asopinae species shows the consistent presence of pointed and long hooks on the mandible, which are absent in seed and stink bugs.

The basic plan of the maxillary stylets constitutes a powerful morphological adaptation revealing external features adapted for tissue penetration and internally includes a delivery channel for saliva and food equipped with small teeth-like/brush-like structures. Generally, the maxillary tips of heteropteran species are sharp, but this varies between taxa [1,2,7,8,14,15,16,17,18,19,20,21,22,66,67] depending on feeding habits. A common feature in most heteropterans the asymmetric tips of the left and right maxillary stylets (curvature on the right and straight on the left) and the distal end of each maxilla being smooth [1,8,15], a feature also visible in asopine species. This analysis of the maxillary stylets showed some differences among three Asopinae species (*P. bidens*, *P. lewisi,* and *C. bhoutanica*). In *P. bidens* and *P. lewisi*, the number of inner teeth is 5, with 2 in *C. bhoutanica*. In these species, the right maxilla is straight with ventral rows that have short barbs in the food canal. Images of the maxillae of related predaceous heteropterans [1,2,8,14,15,17,21,66,67] indicate that the distal parts of the maxillary stylets have different types of barbs consisting of rows of very well-developed stiff bristles in the food canal. A system of barbs in the food canal of predaceous heteropterans is needed to filter larger solid food so that they do not obstruct the digestive tract [1,8]. However, our observations of Asopinae show very few stiff bristles in the canal compared to other predatory insects [1,15,21].

### 4.2. Sensilla Types

Usually, the labium surface of heteropteran insects bears different sensilla with functions that vary between mechanoreceptive, chemoreceptive (gustatory and olfactory), proprioceptive, and thermo-hygrosensitive [1,15,16,18,19,20,24,26,27,28,68,69]. Here, we focus on comparing three predatory stinkbugs with other asopine species as well as with phytophagous pentatomid bugs.

The morphological characteristics of sensilla in Asopinae have been little studied. Basic information the labial tip sensilla and sensilla located around the distal part of last labial segment described in *Canthecona furcellata* [28] and in *Eocanthecona furcellata* [26]. More data on the labial sensilla were presented for *Perillus bioculatus* and *Eocanthecona furcellata* [27]. A full analysis of sensilla on each labial segment in other species indicates that up to fourteen different morphological types of sensilla may be present. In general, the basic shapes of labial sensilla are morphologically similar to sensilla reported for other heteropteran insects, including asopine species [26,27,28]. Nevertheless, in *P. bidens*, *P. lewisi,* and *C. bhoutanica* a new characteristic of sensilla was also distinguished. Our observations of the sensilla (St1) on the labial surface show that the number, types and distribution are similar between the two Picromerus species (*P. bidens* and *P. lewisi*) but lacking in *C. bhoutanica*. In the latter species the sensilla basiconica Sb3 are analogous to St1 because both have a similar system of pores. The short sensilla trichodea (St1) and sensilla basiconica Sb3 with several cuticular (nano) pores (in approximate diameters of St1 = 58.7 ± 4.3 nm, Sb3 = 31.6 ± 2.0 nm) were observed on the surfaces of segments 1–4; however, these sensilla are rarely arranged on the labium. Nanopores are small (1–100 nm diameter) holes or channels formed in biological membranes [70]. The permeation of ions and small molecules through nanopores is common in biological systems [70]. We suggest that these sensilla St1 and Sb3 might perform some kind olfactory function, because they have a special channel for uptake of nanoparticles. The ability to smell airborne odorants contributes to the insect’s ability to search for food and mates and detect environmental cues. In insects, the cuticle covering sensilla has small pores with diameters between 50 and 200 nanometers [70]. These nanopores are believed to function as filters that allow odorant molecules to enter but prevent the entry of larger airborne particles and help the insects avoid liquid loss [70]. Cuticular nanopores were described in the fruit fly’s olfactory sensilla [70]. The presence of pores on the wall of the sensillum is characteristic of olfactory sensilla [5,54,55]. These types of sensilla have not been reported in other predatory stink bugs [26,27,28]. However, a recent study on *Haematoloecha nigrorufa* (Reduviidae: Ectrichodiinae) [21] that employed very high magnification did provide the first evidence of the presence of St1 with pores in a predaceous heteropteran. As such types of sensilla are similar to antennal sensilla and are reported to have an olfactory function [5,71,72], it seems possible that the hemipteran labium and probably the labrum assist the antenna in this function.

The sensilla trichodea II (St2) are smooth, slender, and inserted in a flexible socket in the subapical region of the labium in all three species (*P. bidens*, *P. lewisi* and *C. bhoutanica*) and appear to be identical to those of other heteropterans also in Asopinae [16,18,19,20,26,27,28]. Their basic morphological appearance corresponds with the features of mechanosensilla also reported in other insects [54,55].

Observations of sensilla basiconica (Sb1, Sb2, Sb5, Sb6) on the labium in *P. bidens*, *P. lewisi*, and *C. bhoutanica* show obvious similarities. Only slight differences in the size and distribution of these sensilla were observed (Table 2); however, this may be attributed to the individual characteristics of the studied species. These sensilla basiconica are robust and have a flexible socket. This structure indicates a mechanical function, and they appear to be identical to those of the asopines studied by Rani [26] and Parveen et al. [27]. The sensilla campaniformia (Sca1, Sca2) distributed in various places on the mouthparts in Asopinae, in phytophagous pentatomids as well as in other heteropterans [16,18,19,20,27,28,73,74] represent the same form and function. These are mechanosensilla with a function of proprioception, responding to strains in the exoskeleton [75,76]. Similar functions are performed by sensilla basiconica (Sb4) described in this study. These are present on the junction between the first and second labial segment, and between the third and fourth segment. Such sensilla are also present in other heteropterans [16,18,19,20]. Proprioceptive sensilla perceive the degree of flexion of the integument [55,77].

The comparison of the labial tip sensilla of the studied species with other Asopinae species (*Eocanthecona furcellata* by Rani [26] and Parveen et al. [27], *Perillus bioculatus* by Parveen et al. [27] and *Canthecona furcellata* by Barsagade and Gathalkar [28] reveals a few differences in distribution of various sensilla types. This study of three species revealed nine pairs of sensilla styloconica (Sst) (nos. 1–6, 8–10), similar to gustatory sensilla corresponding to sensilla basiconica type A described in *Eocanthecona furcellata* by Rani [26], to sensilla styloconica (SStc), and peg sensilla (SP1) in *Perillus bioculatus* and *Eocanthecona furcellata* reported by Parveen et al. [27], and to sensilla basiconica (SB-I) in *Canthecona furcellata* reported by Barsagade and Gathalkar [28]. Two pairs of sensilla basiconica VII (Sb7) (nos. 7, 11 are the same type of gustatory sensilla) are short and have a big terminal pore; these pegs are located among sensilla styloconica. Sb7 are similar to sensilla basiconica type B and C [26] and to peg sensilla (SP2) in all five species studied by Parveen et al. [27], and to sensilla basiconica (SB-II, SB-III) in *Canthecona furcellata*. According to Rani [26] the type C and SB-III distinguished by Barsagade and Gathalkar [28] are only slightly different from each other because they are surrounded by hair-like structures. Rani [26] suggested that because sensilla basiconica type B and C have wall pores, they may have an olfactory function. Barsagade and Gathalkar [28] did not attribute specific functions to the sensilla reported. In this study, the system pores of the sensilla basiconica (Sb7) are not visible, although a large magnification was used in SEM observations. We agree with Parveen et al. [27] that sensilla (Sb7) are chemosensilla with gustatory function (uniporous sensilla). Although typical olfactory sensilla are not found on the labial tip in the Asopinae species, the reception of olfactory stimuli probably takes place through uniporous sensilla (gustatory). The uniporous sensilla present on the labial tip, e.g., near the rostral groove opening, might perceive the volatile chemicals emanating from prey. According to Zacharuk [5], all types of uniporous sensilla that respond to gustatory stimuli can also respond to strong odors. Thus, olfactory stimuli can be conducted even in the absence of typical multiporous olfactory sensilla. Sensilla styloconica are restricted to the predatory bugs, i.e., *E. furcellata* and *P. bioculatus* [27] and are located more marginally on the sensory fields. In phytophagous stinkbugs (*Dolycoris indicus* (Stål), *Piezodorus hybneri* (Gmelin) and *Plautia crossota* (Dallas)) uniporous peg sensilla (SP1) are more numerous on the sensory fields.

Sensilla trichodea (St3, St4) on the apical and subapical region of the labium in Asopinae species probably represent mechano-chemosensilla. Their appearance and location are similar to those reported as having this dual function in other insects [54,76,78]. The counterpart mechano-chemosensilla (St3, St4) are sensilla trichodea embedded in a flexible socket with cuticular walls smooth and a terminal pore, that are observed in phytophagous as well as predatory stinkbugs as three subtypes—sensilla trichodea ST1, ST2 and ST3. Sensilla trichodea were more abundant near the labial tip in such a way that they make first contact with the substratum during feeding [27,28]. A similar three subtypes of sensilla trichodea A, B and C were arranged around the labial tip as well as the sensorial area of the labial apex in *E. furcellata* and *C. furcellata* [26,28].

In all mentioned Asopinae species, there are special structures near the rostral groove referred to as cuticular projections (types i, ii, iii) by Parveen et al. [27] or multilobed sensillae (MLS) by Barsagade and Gathalkar [28] and Rani [26]. In our studied species, these structures are visible Figure 9. They apparently lack sensillar structures, although short and smooth peg sensilla with thermo-hygroreceptive function may be hidden among them. This type of sensillum is common in heteropteran species [16,18,19,20,24,27,69] and other insects [79].

## 5. Conclusions

The mouthpart structures of three Asopinae species were investigated as examples of predatory stink bugs. Our results represent the first detailed reports of *P. bidens*, *P. lewisi*, and *C. bhoutanica* mouthpart structures. These species exhibit some similar structures compared to other stink bugs: similar labium shape but different lengths and similar basic types of sensilla on the labial surface.

The sensilla on the labium surface (especially several types of sensilla basiconica Sb1, Sb2, Sb4, Sb6) in these three species are not significantly different in terms of their presence and location. In general, the labial tip sensilla in Asopinae reflects the usual pentatomorphan pattern. However, in these predators the sensilla styloconica are predominant along with additional short and long cuticular projections that are numerous around these sensilla. The two types of olfactory sensilla with nanopores (St1, Sb3) probably function to locate prey by smell.

In Asopinae, adaptation to the predatory lifestyle contributed to the formation of several hook-shaped mandibular teeth, clearly different from homologous structures in phytophagous stinkbugs. Externally, however, the structures of the maxillary stylets do not show significant changes reflecting a shift from phytophagy to predation; rather the phytophagous characters are preserved. The presence of distinct, stiff bristles in the food canal may indicate a possible adaptation to feeding on insect larvae.

## Figures and Tables

**Table 1:** Comparison of the lengths of labial segments in studied species. Data are means ± SE values obtained from scanning electron microscopy.

Species	Labrum	Labial Segments				
		I (μm)	II (μm)	III (μm)	IV (μm)	Whole
*Picromerus bidens* (Linnaeus)	1586.8 ± 10.6	967.3 ± 8.9	1527.7 ± 33.2	1080.5 ± 34.5	1113.4± 24.4	4687.8 ± 93.9
*Picromerus lewisi* Scott	1694.8 ± 55.9	1046.0 ± 12.1	1474.1 ± 74.2	1068.3 ± 9.2	1073.9 ± 17.8	4661.5 ± 89.2
*Cazira bhoutanica* Schouteden	1385.9 ± 2.9	862.1 ± 7.4	1379.1 ± 28.6	925.2 ± 7.4	1001.2 ± 12.5	4179.8 ± 43.1

**Table 2:** Distribution and morphometric data of sensilla in studied species. Data are means ± SE values obtained from scanning electron microscopy. N = sensilla number; Lm, Labrum; St1–4, sensilla trichodea I–IV; Sb1–7, sensilla basiconica I–VII; Sst, Sensilla styloconica; Sca1–2, sensilla campaniformia I–II; Lb, labium; Lb1, 2, 3, 4, the first, second, third, fourth segment of labium; SF, sensory field on the labial tip.

Sensilla	*Picromerus bidens* (Linnaeus)				*Picromerus lewisi* Scott				*Cazira bhoutanica* Schouteden			
	Distri- bution	Length (μm)	Basal Diameter (μm)	N	Distri- bution	Length (μm)	Basal Diameter (μm)	N	Distri- bution	Length (μm)	Basal Diameter (μm)	N
St1	Lb1-4	22.9 ± 1.2	0.83 ± 0	10	Lb1-4	16.4 ± 1.5	0.8 ± 0	10				
St2	Lb4	17.3 ± 1.8	1.6 ± 0.1	8	Lb4	20.7 ± 1.1	1.2 ± 0.1	10	Lb4	22.5 ± 2.0	1.7 ± 0.1	7
St3	Lb4	63.5 ± 1.9	2.1 ± 0.1	5	Lb4	66 ± 4.9	2.3 ± 0.1	6	Lb4	97.6 ± 9.8	2.5 ± 0.1	6
St4	SF	15.6 ± 0.8	1.7 ± 0.1	10	SF	15.5 ± 1.1	1.6 ± 0.1	10	SF	4.2 ± 0.3	1.2 ± 0.1	6
Sb1	Lm, Lb1-4	9.4 ± 0.6	1.7 ± 0.1	10	Lm, Lb1	12 ± 1.8	1.9 ± 0.1	10	Lm, Lb1	9.2 ± 0.7	1.5 ± 0.1	9
Sb2	Lb1	67.8 ± 9.5	3.8 ± 0.2	5	Lm, Lb2	38 ± 5.9	3.7 ± 0.6	7	Lb1, 2	56.2 ± 4.6	4.3 ± 0.2	4
Sb3					Lm	1.7 ± 0.6	0.7 ± 0	3	Lm, Lb1-3	1.1 ± 0.1	0.8 ± 0.1	3
Sb4	Lb2, Lb4	23.3 ± 0.7	4.2 ± 0.1	5	Lb2, Lb4	18.3 ± 0.6	3.6 ± 0.1	4	Lb2, Lb4	11.2 ± 1.3	3.5 ± 0.2	4
Sb5	Lb1, Lb3	14.9 ± 0.4	1.8 ± 0.1	3	Lb1,2,4	8.7 ± 0.7	1.4 ± 0.1	4	Lb2	10.9 ± 1.0	1.8 ± 0.1	5
Sb6	Lb1-4	18 ± 1.4	1.5 ± 0.1	8	Lb3,4	40.3 ± 0.4	3.5 ± 0.4	3	Lb2-4	75.3 ± 3.9	4.1 ± 0.1	3
Sb7	SF	4.7 ± 0.4	2.6 ± 0.1	4	SF	4.4 ± 0.8	1.4 ± 0.1	4	SF	4.9 ± 0.2	2.5 ± 0.1	4
Sst	SF	22.8 ± 0.9	7.3 ± 0.2	18	SF	22.8 ± 0.5	7.5 ± 0.3	10	SF	11.6 ± 0.8	5.1 ± 0.2	9
Sca1	Lm		7.1 ± 0.5	4	Lm, Lb2		6.7 ± 0.2	3	Lm, Lb2, 4		9.1 ± 0.9	3
Sca2	Lb4		5.5 ± 0.3	12	Lb4		5.8 ± 0.7	10	Lb4		7.4 ± 0.4	4

**Table 3:** Terminology and definition of sensilla used in this paper including terms from previous studies [27,54,55], Wp, wall pore; Tp, tip pore.

Type	Shape	Socket	Surface	Pore	Category	Function
St1	Hair in pit	Inflexible	Smooth	Wp (porous) diameter 0.043 (μm) = 43 nm	Chemoreceptive sensilla	Olfactory
St2	Hair	Flexible	Smooth	No	Mechanoreceptive sensilla	Tactile
St3	Hair	Flexible	Smooth	No	Mechanoreceptive sensilla	Tactile
St4	Hair	Inflexible	Smooth	No	Mechanoreceptive sensilla	Tactile
Sb1	Peg	Flexible	Grooved	No	Mechanoreceptive sensilla	Tactile
Sb2	Peg	Flexible	Grooved	No	Mechanoreceptive sensilla	Tactile
Sb3	Peg in pit	Inflexible	Smooth	Diameter 0.043 (μm) = 43 nm	Chemoreceptive sensilla	Olfactory
Sb4	Peg	Flexible	Smooth	Wp (molting pore)	Proprioceptive sensilla	Perceive the degree of flexion of the joint
Sb5	Peg	Flexible	Smooth	No	Mechanoreceptive sensilla	Tactile
Sb6	Peg, hair	Flexible	Grooved	No	Mechanoreceptive sensilla	Tactile
Sb7	Peg in pit	Inflexible	Smooth	TP	Chemoreceptive sensilla	Gustatory
Sst	Peg	Cone sitting on a style(high socket)	Smooth	TP	Chemoreceptive sensilla	Gustatory
Sca1	Oval plate	Inflexible	Smooth	Central pore (molting pore)	Mechanoreceptive sensilla	Responding to strains in the exoskeletons
Sca2	Domelike structures	Inflexible	Smooth	Central pore (molting pore)	Mechanoreceptive sensilla	Responding to strains in the exoskeletons

**Table 4:** Main features of mouthparts of stinkbugs (Pentatomidae).

Feeding Behavior	Species	Labrum	Labium	Number of Sensilla Types	The Number of Sensilla of Labium Tip	Apical Plate	Internal Side of Maxillary Stylet	Internal Morphology of Mandibular Stylet	Distal Mandibular Stylet; Serration	References
Predatory	*Canthecona furcellata* (Wolff)	triangular sclerite	elongated	3 types	unreported	unreported	dentations	unreported	serrated	Barsagade & Gathalkar [28]
Predatory	*Eocanthecona furcellata* (Wolff)	unreported	robust and stout	6 types	3 types	unreported	unreported	unreported	unreported	Rani [26]; Parveen et al. [27]
Predatory	*Perillus bioculatus* Fabricius	unreported	robust and stout	6 types	3 types	membranous lobe microtrichial extensions	unreported	squamous texture	sharp recurved hooks	Cobben [1]; Parveen et al. [27]
Predatory	*Podisus maculiventris* (Say)	unreported	unreported	unreported	unreported	present	unreported	unreported	teeth	Cohen [8]
Predatory	*Picromerus bidens* (Linnaeus)	elongated, triangular	robust and stout	14 types	3 types	membranous lobe microtrichial extensions	short barbs	squamous textures and cuticular spines	five teeth and three hooks	This study
Predatory	*Picromerus lewisi* Scott	elongated, triangular	robust and stout	14 types	3 types	membranous lobe microtrichial extensions	short barbs	squamous textures and cuticular spines	five teeth and three hooks	This study
Predatory	*Cazira bhoutanica* Schouteden	elongated, triangular	robust and stout	14 types	3 types	membranous lobe microtrichial extensions	short barbs	squamous textures and cuticular spines	five teeth and three hooks	This study
Phytophagous	*Graphosoma lineatum* (Linnaeus)	unreported	unreported	unreported	unreported	unreported	unreported	small, widely spaced notches arranged in longitudinal strips	complex ribbed texture, the apices are knotted with irregular prominences	Cobben [1]
Phytophagous	*Chrysocoris purpurea* (Westwood)	unreported	unreported	unreported	2 types	present	unreported	unreported	knotted with irregular prominences	Rani & Madhavendra [25]
Phytophagous	*Cyclopelta siccifolia* Westwood	unreported	unreported	unreported	2 types	present	unreported	unreported	knotted with irregular prominences	Rani & Madhavendra [25]
Phytophagous	*Dichelops melacanthus* (Dallas)	unreported	unreported	unreported	unreported	unreported	unreported	squamous texture	dentition	Depieri & Panizzi [12]
Phytophagous	*Dolycoris indicus* (Stål)	unreported	slender and long	6 types	2 types	unreported	unreported	unreported	unreported	Parveen et al. [27]
Phytophagous	*Euschistus heros* Fabricius	unreported	unreported	unreported	unreported	unreported	unreported	squamous texture	dentition	Depieri et al. [13]; Depieri & Panizzi [12]
Phytophagous	*Nezara viridula* (Linnaeus)	unreported	unreported	unreported	2 types	present	grooves	squamous texture	knotted with irregular prominences	Rani and Madhavendra [24]; Depieri & Panizzi [12]
Phytophagous	*Piezodorus guildinii* (Westwood)	unreported	unreported	unreported	unreported	unreported	unreported	squamous texture	dentition	Depieri & Panizzi [12]
Phytophagous	*Piezodorus hybneri* (Gmelin)	unreported	slender and long	6 types	2 types	unreported	unreported	unreported	unreported	Parveen et al. [27]
Phytophagous	*Plautia crossota* (Dallas)	unreported	slender and long	6 types	2 types	unreported	unreported	unreported	unreported	Parveen et al. [27]
